# NNICE: a deep quantile neural network algorithm for expression deconvolution

**DOI:** 10.1038/s41598-024-65053-w

**Published:** 2024-06-18

**Authors:** Yong Won Jin, Pingzhao Hu, Qian Liu

**Affiliations:** 1 Department of Biochemistry & Medical Genetics, Rady Faculty of Health Sciences, University of Manitoba, Winnipeg, MB R3E 0J9 Canada; 2 Department of Biochemistry, Schulich School of Medicine & Dentistry, Western University, London, ON N6A 5C1 Canada; 3 Department of Applied Computer Science, University of Winnipeg, Winnipeg, MB R3B 2E9 Canada

**Keywords:** Computational biology and bioinformatics, Immunology

## Abstract

The composition of cell-type is a key indicator of health. Advancements in bulk gene expression data curation, single cell RNA-sequencing technologies, and computational deconvolution approaches offer a new perspective to learn about the composition of different cell types in a quick and affordable way. In this study, we developed a quantile regression and deep learning-based method called Neural Network Immune Contexture Estimator (NNICE) to estimate the cell type abundance and its uncertainty by automatically deconvolving bulk RNA-seq data. The proposed NNICE model was able to successfully recover ground-truth cell type fraction values given unseen bulk mixture gene expression profiles from the same dataset it was trained on. Compared with baseline methods, NNICE achieved better performance on deconvolve both pseudo-bulk gene expressions (Pearson correlation R = 0.9) and real bulk gene expression data (Pearson correlation R = 0.9) across all cell types. In conclusion, NNICE combines statistic inference with deep learning to provide accurate and interpretable cell type deconvolution from bulk gene expression.

## Introduction

The composition of cell types and their proportions in tissues are key indicators of people’s health. Take the immune cell types as examples, in most cancers, higher levels of T lymphocytes have been associated with favourable clinical outcomes^1,2^, whereas macrophages have been known to be pro-tumourigenic^3^. Traditionally, experimental approaches such as conventional histology procedures and contemporary flow cytometers are commonly used to determine the diversity and composition of cell types within a bulk tissue sample. However, recently, advancements in bulk gene expression data curation, single cell RNA-sequencing (scRNA-seq) technologies, and computational deconvolution approaches have all come together to offer a new perspective to learn about the composition of different cell types in a quick and affordable way.

Today, there is an abundance of bulk transcriptomic data available publicly online^4^. These bulk transcriptomes often represent the average gene expression across a heterogeneous mixture of cells. If cellular components and their proportions can be identified from bulk transcriptomic data by computational methods, such in-silico methods can be used to characterize and quantify immune infiltrates in a cost-, time-, and labor-effective manner. Deconvolution is a computational problem of simplifying a complex mixture into its individual constituents. In brief, most deconvolution algorithms see bulk transcriptome as a mixture where one gene of the mixture is a linear combination of that gene expressed across different cell types, weighted by the proportions of those cell types^5^.

There are numerous expression deconvolution tools that have been developed over the recent decades; however currently, there is not a single “gold-standard” tool for expression deconvolution^5,6^. A main challenge with research in expression deconvolution is that these studies require an interdisciplinary approach with considerations for two ever-changing fields in science: machine learning, and molecular biology. Just within the last few decades, genomics research has already gone through several technological revolutions starting with the microarrays, then to next-generation sequencing, and most recently scRNA-seq technologies^7^. On the other hand, the machine learning field has advanced just as fast with developments in artificial intelligence—most notably deep learning and artificial neural network (ANN)-based algorithms^8^. Despite the huge success of ANN algorithms in computer vision and recently in bioinformatics, only a few studies have explored how such algorithms should be applied to the problem of expression deconvolution. A recently developed tool based on ANN called digitalDLsorter was trained on simulated pseudo-bulk data generated from scRNA-seq datasets and produced immune fraction estimates that correlated with estimates from other methods as well as providing prognostic values in breast and colorectal cancer patients^9^. Another recent ANN-based tool, Scaden, was trained rigorously on both simulated and real gene expression profiles (GEPs), demonstrating robust performances in recovering immune cell type fractions, even against existing methods^10^. However, these tools did not consider their uncertainty as much as their accuracy, which is often critical for many real-world problems.

**Figure 1:**
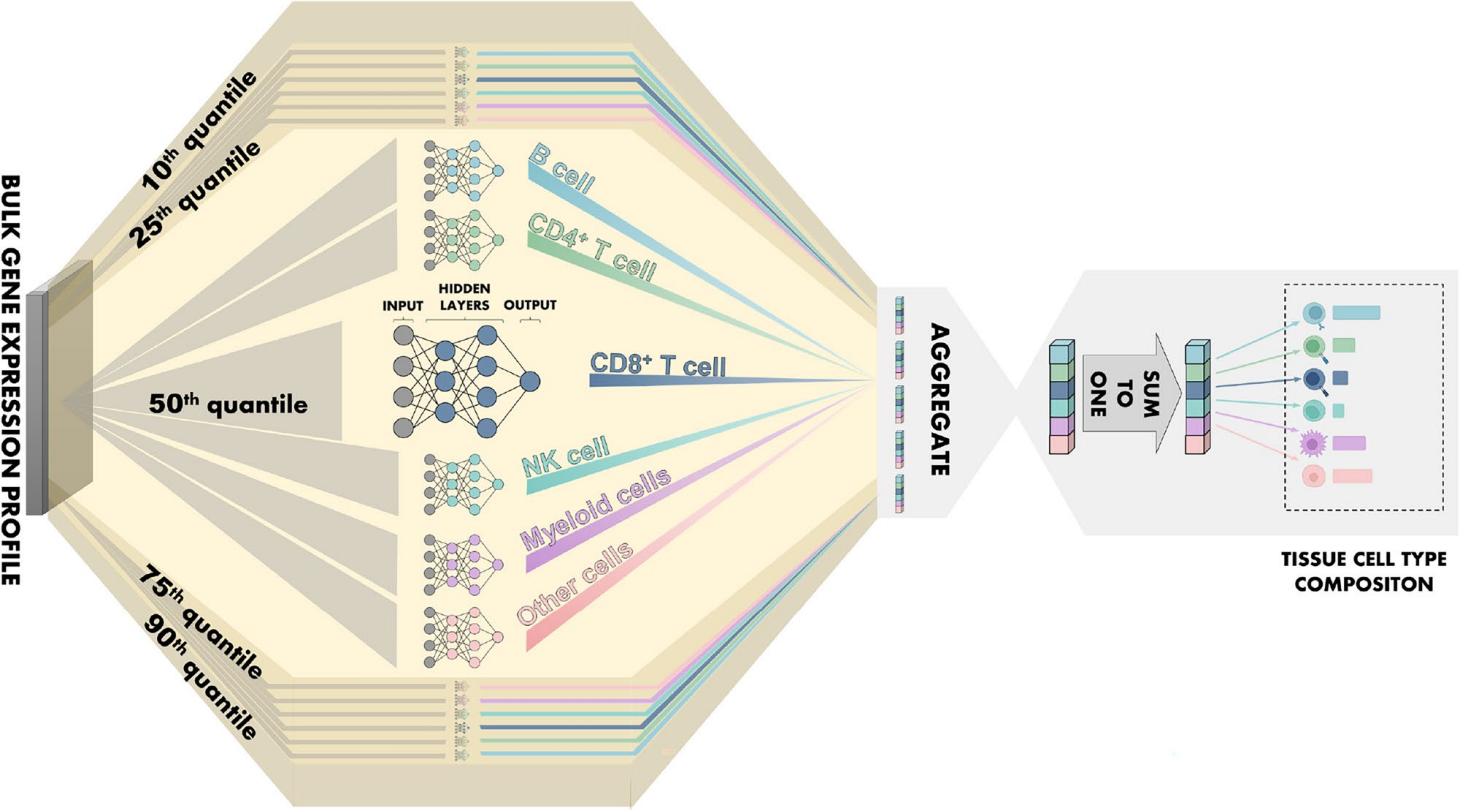
Illustration of Neural Network Immune Contexture Estimator (NNICE) expression deconvolution model. Different quantiles (10%, 25%, 50%, 75%, 90%) were selected from the bulk gene expression profile. Each of these quantiles was used to train independent ANNs for each cell type of interest (B cells, CD4^+^ T cells, CD8^+^ T cells, myeloid cells, NK cells, and other cells). Features from these ANNs were aggregated and input into the final expression deconvolution model for cell type composition prediction.

In this study, we present and explore the application of an approach referred to as deep quantile regression (DQR) by fitting the predicted values on a number of conditional quantiles^11^. This allows for visualization of the range of uncertainty, or the confidence interval of the cell type abundance estimates generated by the trained model. Our implementation of DQR involved fitting an independent ANN for each of the selected (10%, 25%, 50%, 75%, 90%) quantiles. This allowed for the visualization of uncertainty in the cell type abundance estimates provided by the model. Multicollinearity is a major obstacle for expression deconvolution models since it is possible for several input features to be correlated with abundance of multiple cell types. For example, *CD3D* is part of the T-cell receptor complex and can be expressed across multiple immune cell types such as cytotoxic CD8^+^ T cells and in diverse subsets of CD4^+^ T cells^12^. Researchers have tried to minimize this problem by regularization^13^, recursive tree-like procedure^14^, and a heuristic strategy^6,15^. To combat potential bias and dropouts from collinearity, we trained separate instances of ANN models for each cell type of interest. The main model to be discussed in subsequent sections will be referred to as “NNICE”—Neural Network Immune Contexture Estimator; refer to Fig. 1 and Algorithm 1.

**Figure:**
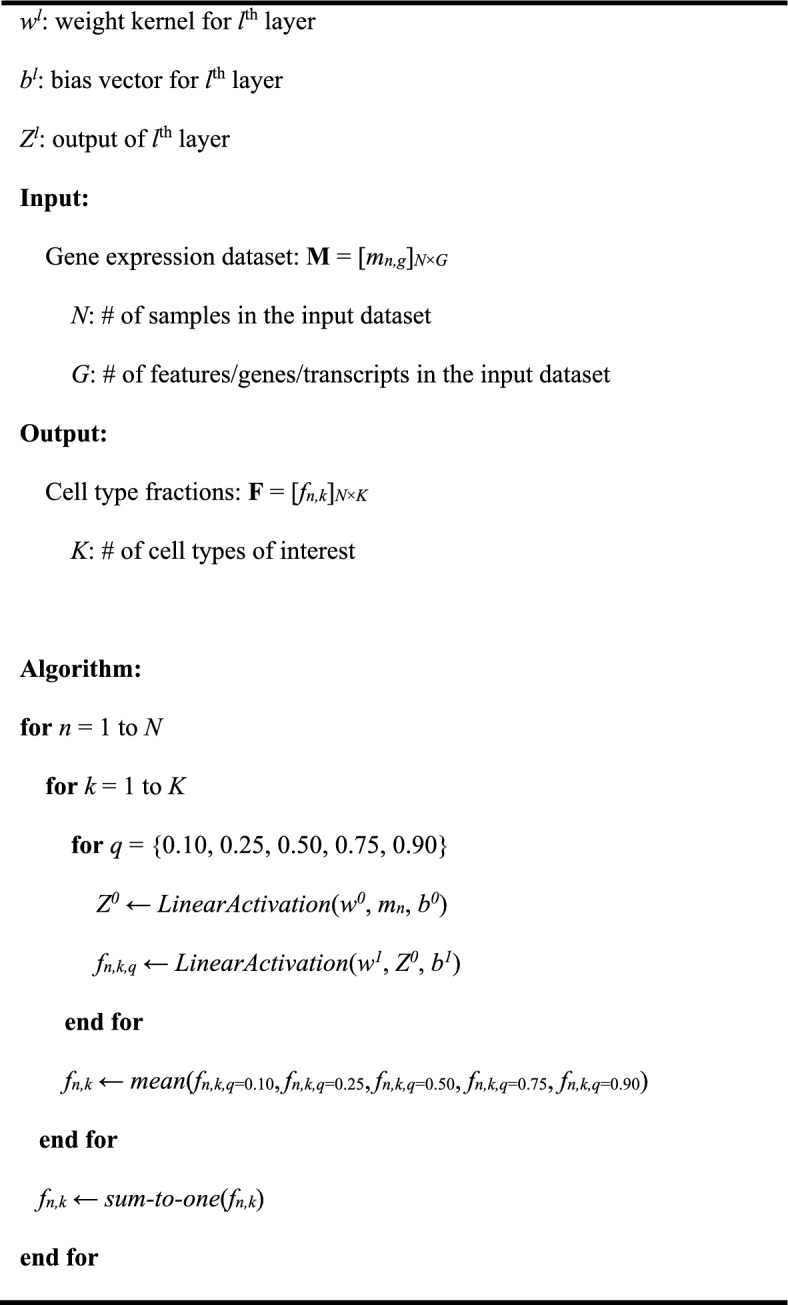
**Algorithm 1** Pseudocode for NNICE expression deconvolution.

## Methods

### Single cell RNA-sequencing data of immune cells

scRNA-seq datasets from immune cells isolated by fluorescence-activated cell sorting (FACS) was downloaded from the 10X Genomics website^12^. Specifically, datasets for the following 10 immune cell types were curated (Table 1): CD14^+^ monocytes, CD19^+^ B cells, CD34^+^ cells, CD4^+^ helper T cells, CD4^+^/CD25^+^ regulatory T cells, CD4^+^/CD45RA^+^/CD25^−^ naïve T cells, CD4^+^/CD45RO^+^ memory T cells, CD56^+^ NK cells, CD8^+^ cytotoxic T cells, CD8^+^/CD45RA^+^ naïve cytotoxic T cells.

**Table 1:** List of scRNA-seq data curated from the 10 × genomics database.

Cell type	Marker	FACS purity	# of cells detected	# of cells after filtering	Reads per cell
B cells	CD19^+^	~ 100%	~ 10,000	9879	~ 25,000
Monocytes	CD14^+^	98%	~ 2,600	2553	~ 100,000
Hematopoietic stem and progenitor cells	CD34^+^	45%	~ 9,000	9080	~ 24,700
Helper T cells	CD4^+^	99%	~ 11,000	11,168	~ 21,000
Regulatory T cells	CD4^+^/CD25^+^	95%	~ 10,000	10,201	~ 27,000
Naïve T cells	CD4^+^/CD45RA^+^/CD25^–^	98%	~ 10,000	10,454	~ 19,000
Memory T cells	CD4^+^/CD45RO^+^	98%	~ 10,000	10,191	~ 24,000
Natural killer cells	CD56^+^	92%	~ 8,000	8238	~ 29,000
Cytotoxic T cells	CD8^+^	98%	~ 10,000	10,187	~ 28,600
Naïve cytotoxic T cells	CD8^+^/CD45RA^+^	99%	~ 12,000	11,891	~ 20,000

**Figure 2:**
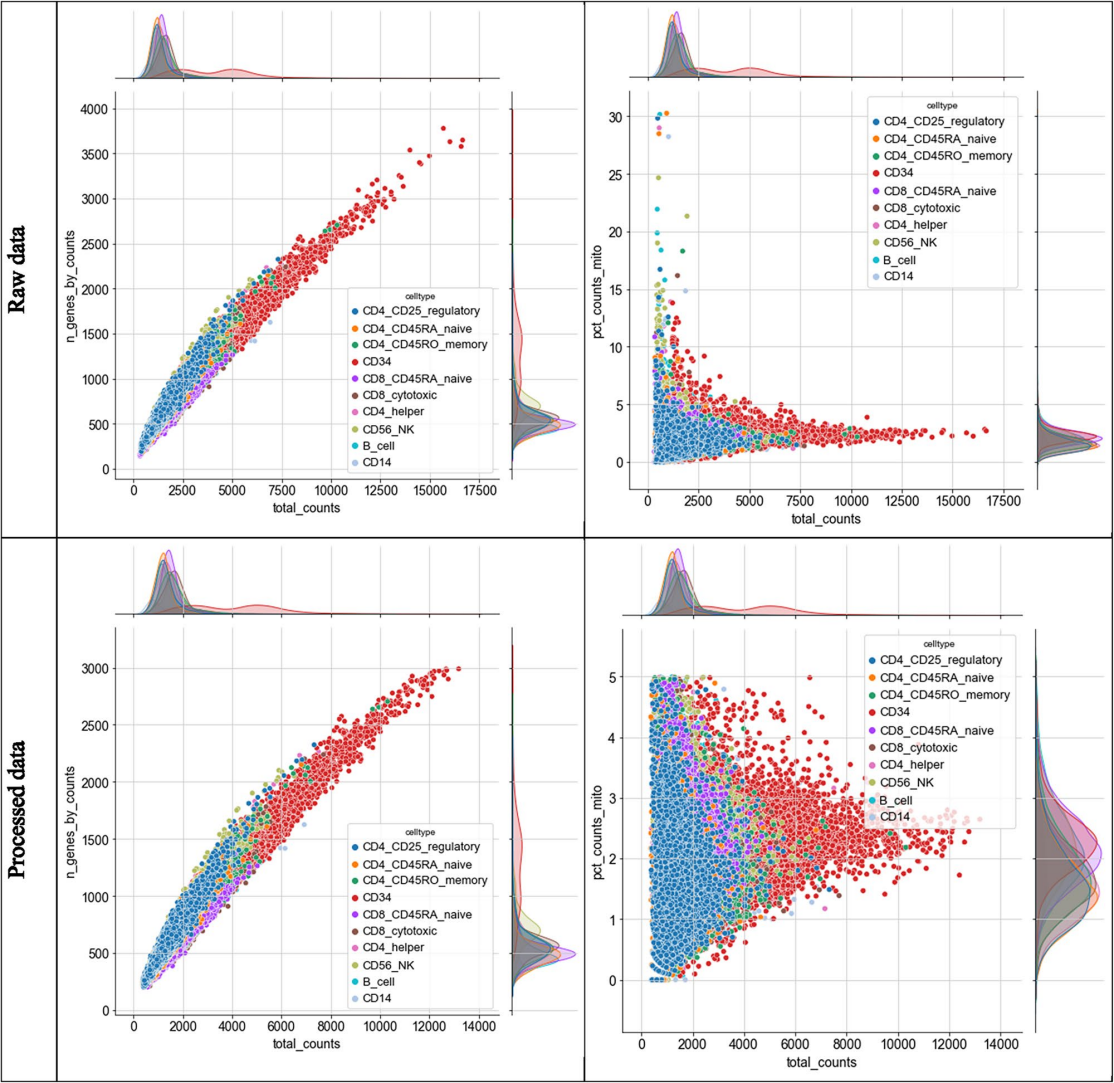
Joint plot of dataset quality control metrics before (top panels) and after (bottom panels) processing the collected scRNA-seq datasets. “n_genes_by_counts” equals the number of genes/features with positive (non-zero) count within the single cell profile. “total_counts” refers to the sum of read counts across all features for a given single cell profile. “pct_counts_mito” refers to the percentage of total read counts that are mitochondrial genes/transcripts.

From the original dataset preprocessed by Cell Ranger 1.1.0, only those cells expressing between 200 and 3000 different features with positive counts, and proportion of total counts from mitochondrial genes under 5% were retained (Fig. 2). Handling of scRNA-seq data was done by using scanpy package^16^ in Python.

**Figure 3:**
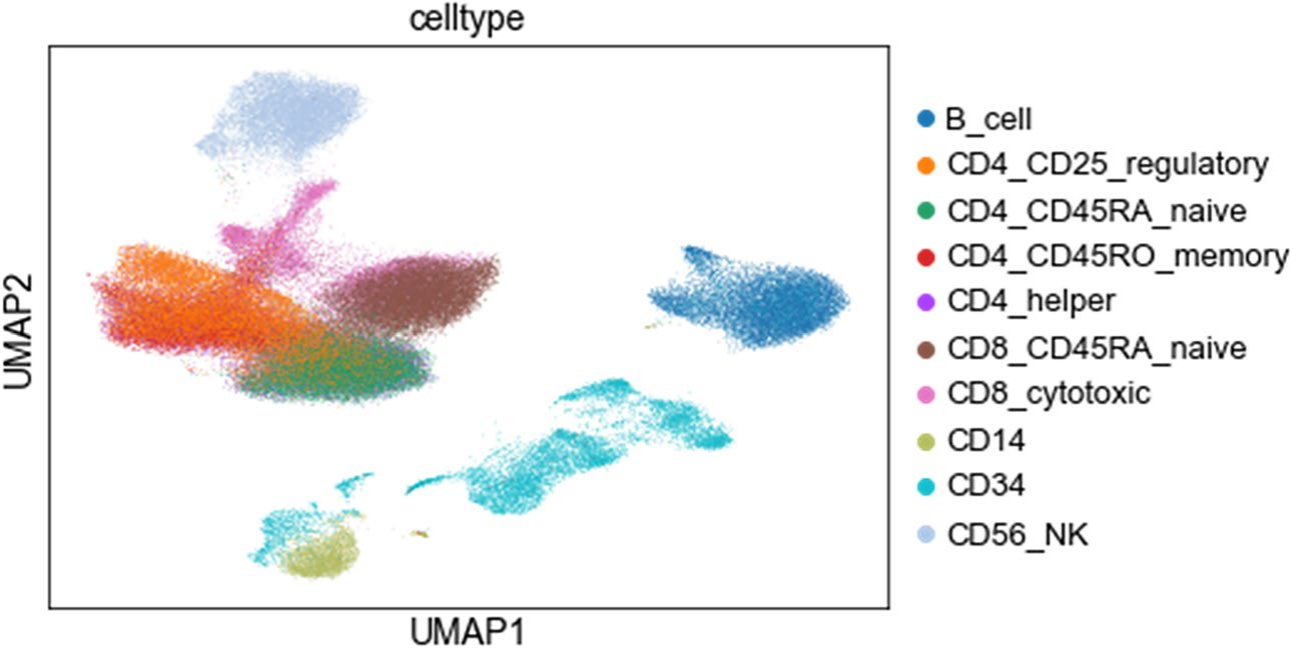
UMAP dimensionality reduction of single cell gene expression profiles into 10 clusters, coloured by the known cell type annotations. The clear separation of clusters suggests that the dataset is robust and that the single-cell gene expression profiles are sufficiently distinct to allow accurate classification by computational algorithms.

To further explore the scRNA-seq dataset, and whether computational algorithms would be able to distinguish single cell GEPs from one cell type to another, uniform manifold approximation and projection (UMAP) algorithm was used to cluster the single cell profiles into 10 groups (Fig. 3).

### Bulk tissue RNA-sequencing data of immune cells with FACS-quantified cell proportions

Bulk GEP data from mRNA-seq (single-end Illumina HiSeq 2000) of PBMCs isolated from 468 whole blood samples obtained from healthy participants aged between 50 and 74 was downloaded from the ImmPort database (accession SDY67) as a read count matrix of 23,113 genes and 468 samples^17^. From here onwards, this dataset will be referred to as “SDY67” dataset. For each sample of whole blood, the cell type proportions quantified by flow cytometry panels was acquired from Monaco et al.^18^. From this dataset, proportions for 14 immune cell types were available: B naïve, B exhausted (Ex), B non-switched memory (NSM), B switched memory (SM), plasmablasts, CD4^+^ T cells, CD8^+^ T cells, basophils, myeloid dendritic cells (mDCs), plasmocytoid dendritic cells (pDCs), natural killer (NK) cells, and the classical (C), intermediate (I) and non-classical (NC) subsets of monocytes^18^. The known proportion of the cells from cell types of interest within each GEP sample will be referred to as “true fractions”. Missing flow cytometry data for specific cell types were filled with zero and given values for each sample were normalized to sum to one by dividing each value by the sample total.

### Immune cell type pooling

**Figure 4:**
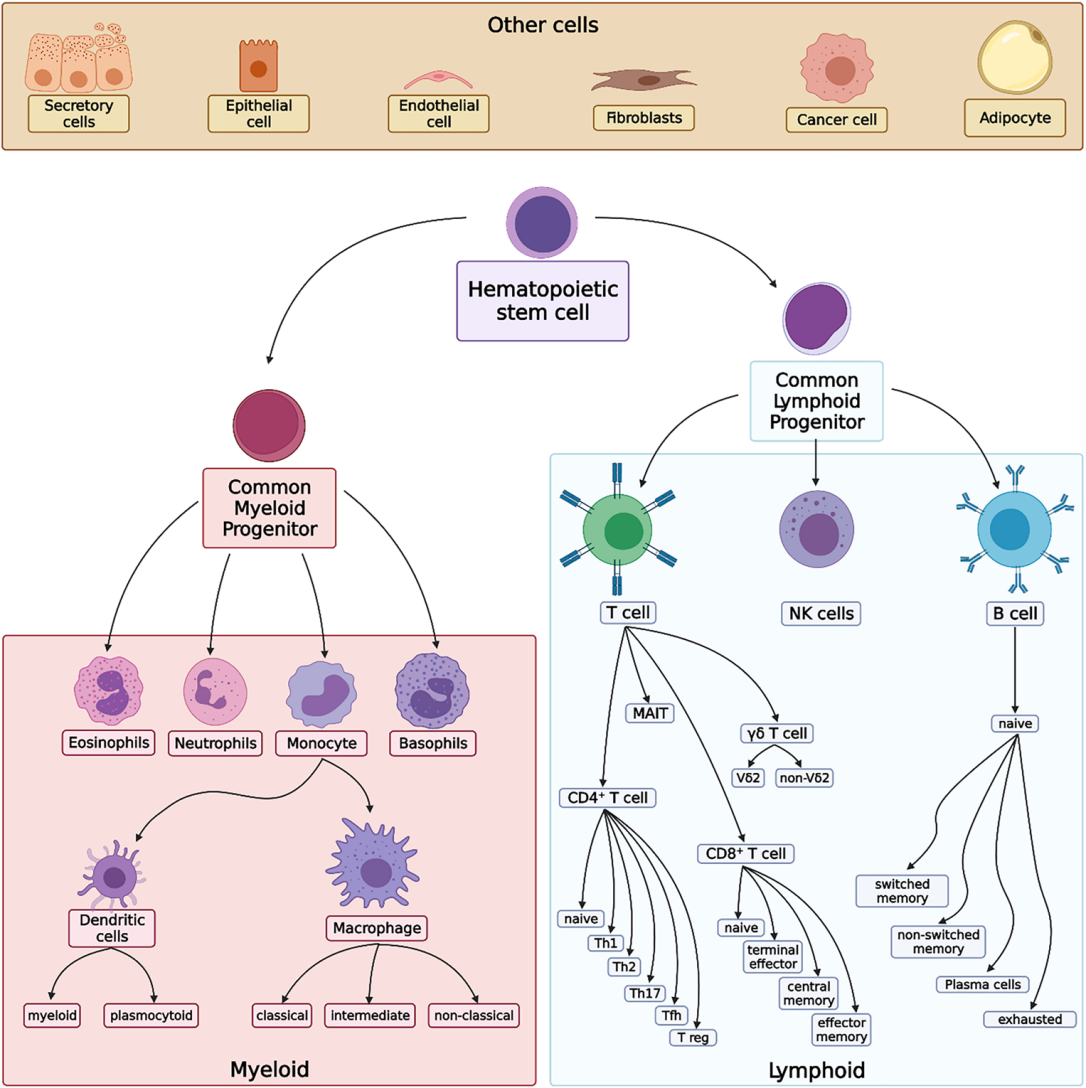
Lineage of immune cells used to pool cell types into broader cell type categories. The illustration shows the hierarchical organization of immune cell development from hematopoietic stem cells to common myeloid and lymphoid progenitors, further differentiating into various immune cell types. The diagram also includes other cells for context.

Since the number of cell types were not the same across datasets (10 for scRNA-seq; 14 for SDY67), the cell types were pooled into the following cell types whenever appropriate: B cells, CD4^+^ T cells, CD8^+^ T cells, myeloid cells, NK cells, and other cells as illustrated in Fig. 4. Cell type lineages were drawn with reference to cell type mappings available in previous studies^5,18^.

### Feature selection

In order for the expression deconvolution model to be applied to a variety of datasets, it was clear from the beginning that there was a need to provide the model with consistent number of input features. Initially, to prevent potential loss of information, all features were provided to the model as input if they were represented consistently across the datasets being used. Interestingly, the model performance did not suffer when only the top 3000 most variable genes selected by SCTransform method^19^ on the scRNA-seq dataset were provided. Furthermore, performance of the trained models significantly improved when the input features were further selected for only those based on the 4815 immunologically related genes from the ImmPort through the InnateDB^20^. Therefore, the final model accepted input features that satisfied all of the following criteria:

1. Included in the ImmPort list of immunologically related features (4,815 genes).
2. Included in all of the datasets used in the study.
3. Top 3000 most variable features determined by SCTransform on scRNA-seq dataset.

Consequently, total of 3000 features were used as input into the final model.

### Pseudo-bulk simulation with known cell type proportions

**Figure 5:**
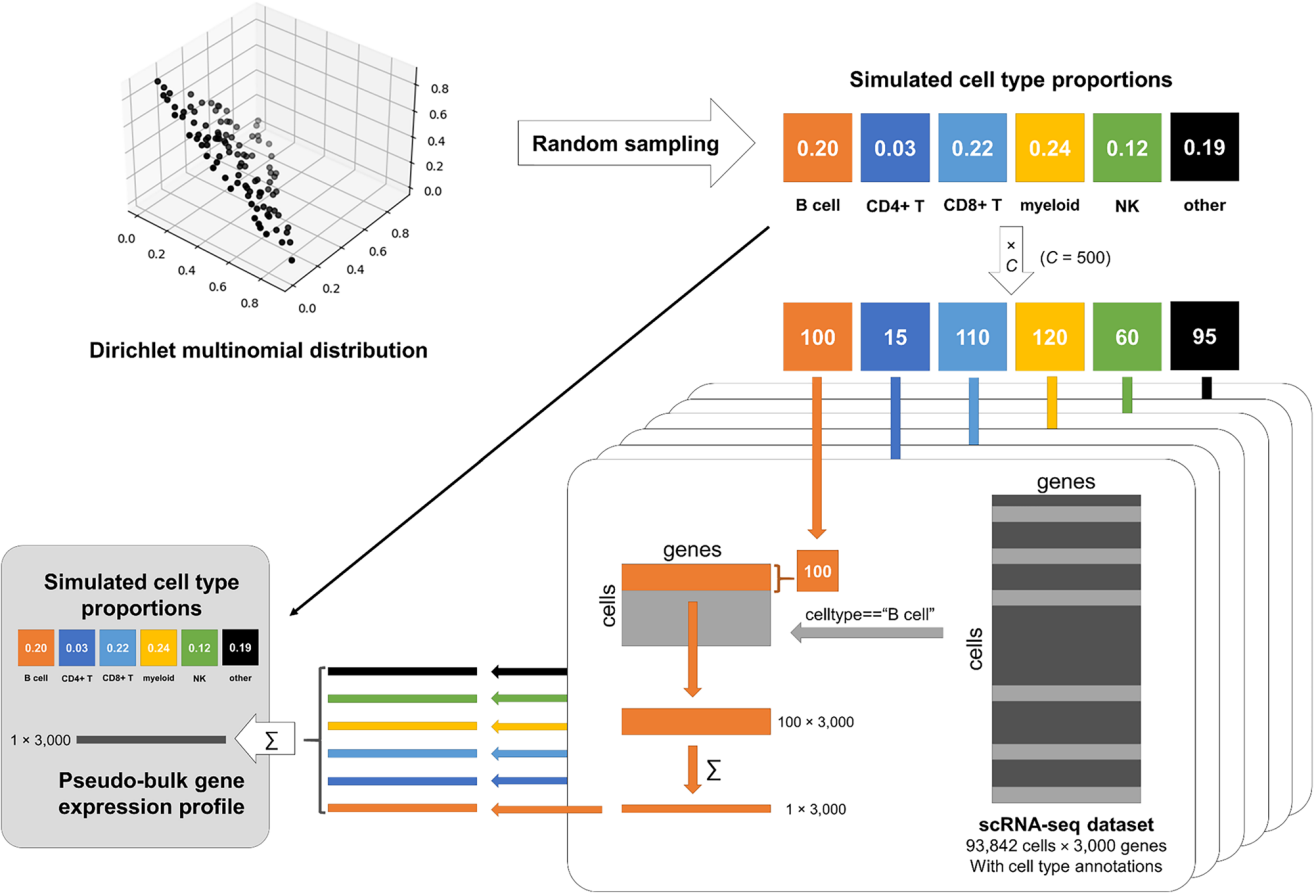
Graphical representation of the pseudo-bulk gene expression profile simulation process using scRNA-seq dataset. The simulation process starts with the Dirichlet distribution sampling, subsampling of cells, and summing their gene expression profiles to create the final pseudo-bulk gene expression profiles.

For the simulation of pseudo-bulk GEPs using scRNA-seq data with G number of features (genes/transcripts), we first ensured that the scRNA-seq samples used for this simulation were distinct from those used in marker selection to prevent information leakage. The cell type fractions for each pseudo-bulk sample were simulated first by sampling vectors of numbers from the Dirichlet distribution for *K* cell types that add up to 1, which represents the proportion of given cell types in each sample. Therefore, *N* by *K* matrix was simulated for *N* pseudo-bulk samples and *K* cell types of interest. Then cells from each of the *K* cell types were randomly subsampled according to the simulated proportion of a total number of cells (*C*) in each pseudo-bulk tissue. Following previous studies that simulated pseudo-bulk GEPs from scRNA-seq data, the total number of cells in each pseudo-bulk sample (*C*) was set to be 500 by default^9,10^. Then the GEPs of *C* subsampled cells were summed together across each feature, resulting in the final pseudo-bulk GEP of 1 by *G*. Graphical summary of this simulation process is shown in Fig. 5.

### Benchmarking against existing expression deconvolution tools

When developing a new computational tool, it is important to compare its performance against existing tools. Five existing expression deconvolution tools available in the immunedeconv^5^ R package were used for performance comparisons against NNICE on benchmark datasets: pseudo-bulk, and SDY67. These existing expression deconvolution tools are listed in Table 2.

**Table 2:** List of existing deconvolution tools used to compare performance against.

Tool name	Acronym	Algorithm	Cell types^a^	Year
CIBERSORT^13^	CBS	ν-SVR	22	2015
TIMER^21^	TMR	Constrained linear least square regression	6	2016
MCP-counter^22^	MCP	Geometric mean of expression marker genes	9	2016
EPIC^23^	EPC	Constrained least square regression	9	2017
quanTIseq^15^	QTS	Constrained least square regression	11	2019

^a^Please note that this number indicates the number of cell types used in the original experiment, not the only number of cell types these methods can handle.

### Neural network model architecture

Recent works in expression deconvolution involving deep learning frameworks used a series of densely connected layers followed by a softmax activation function shown below [Eq. (1)]^9,12^.

1 $$\sigma {(\overrightarrow{z})}_{i}=\frac{{e}^{{z}_{i}}}{\sum_{j=1}^{K}{e}^{{z}_{j}}}$$

Different loss functions for optimizing these models were tested, including root mean square error (RMSE), Pearson correlation loss (1 − R), as well as custom loss function from OnionNet^24^. Custom loss function is defined in Eq. (2).

2 $$\text{Loss}= \alpha \left(1-R\right)+\left(1-\alpha \right)\text{RMSE}$$

Subsequent architectures—named “DNN” models for dense neural network and numbered in chronological order—largely consisted of a series of densely connected layers, linear activation functions, and intermittent batch normalization or drop out layers. Two types of output layers were tested, including softmax activation function and a simple linear activation function with either L1 or L2 regularization.

One way to obtain the range of uncertainty from these models is to fit the predicted values on a number of conditional quantiles^24^. Whereas conventional regression models that predict the mean value of the distribution at each data point are fitted on the residuals (difference against the expected value of the mean) as is, quantile regression can provide the interval between which a percentage of all data points exist within. This is made possible by using the tilted loss function. Given an error metric or residuals (ξ) between true and predicted fractions (ex. Pearson correlation loss (1 − R), root mean square error (RMSE), or custom loss), and q from the set of quantiles {0.10, 0.25, 0.50, 0.75, 0.90}, the tilted loss is represented below as a mathematical expression in Eq. (3).

3 $$\text{TiltedLoss}=max(\left(q\times \xi \right),\left(q-1\right)\times \xi ))$$

During model optimization, it was also realized that instead of training a single model with one input and K outputs for K number of cell types of interest, models were able to provide better estimates when their number of outputs were reduced to a single cell type during optimization. Hence, the final expression deconvolution model was based on DQR and consisted of 6 sub-models, one for each cell type of interest (B cell, CD4^+^ T cell, CD8^+^ T cell, myeloid cells, NK cells, and other cells). Each sub-model consisted of an input layer for 3000 input features connected to a densely connected layer by linear activation functions with 300 neurons, followed by a 1 neuron output layer with linear activation functions with non-negativity constraint on the bias kernel and L2 regularization on the weight kernel with λ = 0.0001. Models were optimized by multiple iterations, or epochs, through a training dataset, minimizing the custom loss function [Eq. (2)] nested inside the tilted loss function [Eq. (3)] across 5 quantiles (0.10, 0.25, 0.50, 0.75, 0.90).

### Model evaluation

For performance metrics, Pearson correlation (*R*) and RMSE were used. Formula for *R* is shown in Eq. (4), where $y$ represents true fractions (labels), $\widehat{y}$ represents estimated fractions, and the bar above variables ($\overline{y }, \overline{\widehat{y} }$) represents the mean of the given vector.

4 $$R=\frac{\sum (\widehat{y}-\overline{\widehat{y} })(y-\overline{y })}{\sqrt{\sum {(\widehat{y}-\overline{\widehat{y} })}^{2}\sum {(y-\overline{y })}^{2}}}$$

For RMSE, the formula is as follows:

5 $$RMSE=\sqrt{\frac{\sum_{i=1}^{n}{({\widehat{y}}_{i}-{y}_{i})}^{2}}{n}}$$

where *n* represents total number of samples in the minibatch.

We first evaluated our model performance using the simulated pseudo-bulk dataset, which consisted of 10,000 training samples and separately simulated 1000 test/validation samples. Next, we evaluated our model performance on the real SDY67 dataset (468 samples). Since there is only limited number of samples from the real datasets, tenfold cross-validation (CV) approach was used to train and validate the model on the SDY67 dataset. This means that a small portion of the dataset (10% in this study) was set aside as internal validation data to validate performance of the model that is trained on a larger subset of the dataset referred to as the training data (90% of samples). Therefore, 10 instances of the model were created—each with trained with 90% of the dataset and tested on a unique remaining subset (10%) of the dataset.

## Results

### Evaluation of model trained on pseudo-bulk GEPs

**Figure 6:**
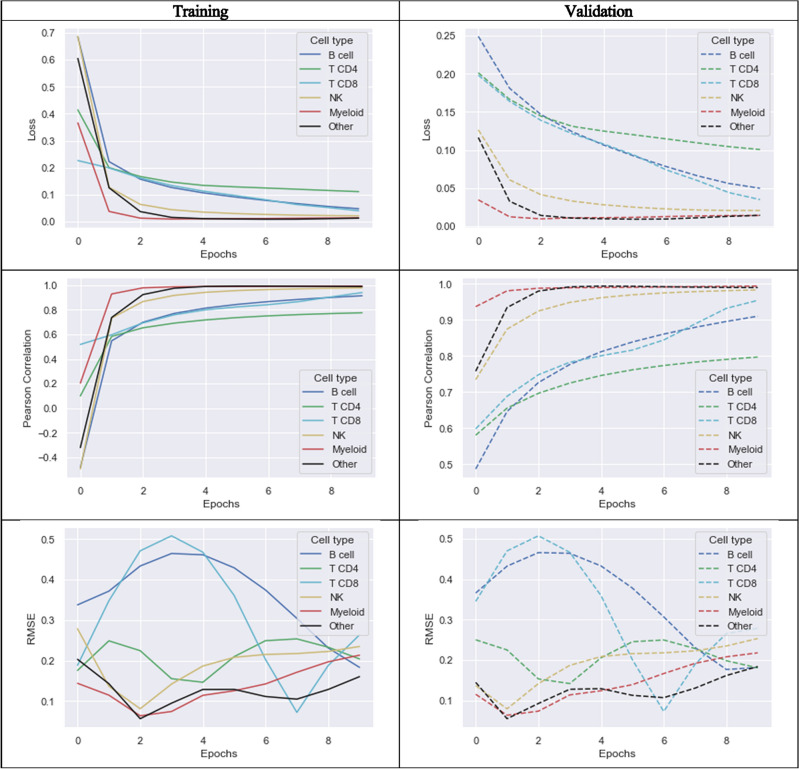
Training and validation history of performance metrics for different cell types. The plots illustrate the performance of the models over epochs for both training and validation datasets. The metrics shown include loss, accuracy, and other relevant performance indicators, separately for each cell type: B cell, T CD4, T CD8, NK, Myeloid, and Other cells. The solid lines represent the training phase, while the dashed lines represent the validation phase.

**Figure 7:**
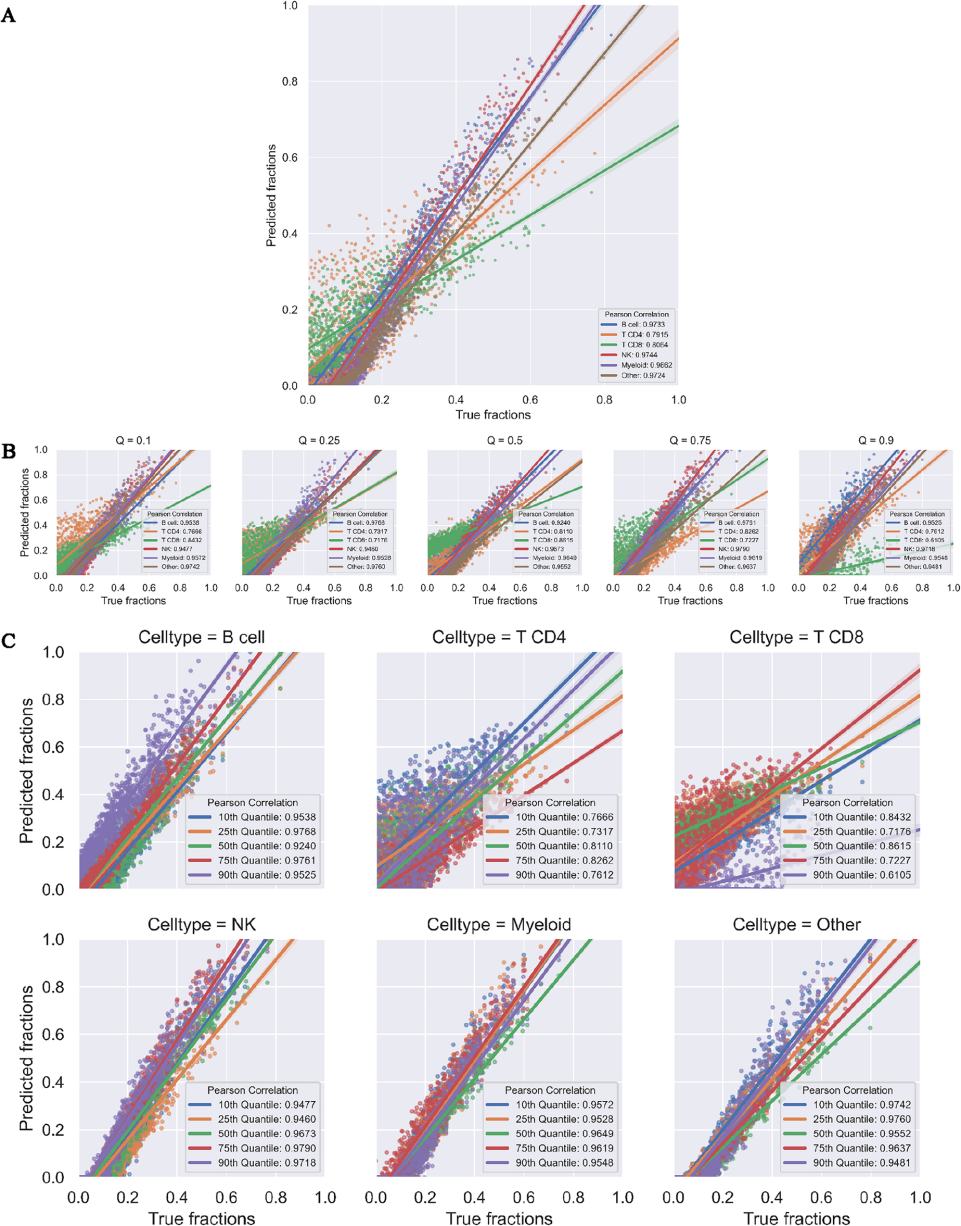
Scatter plots of true cell type fractions (x-axis) by fractions predicted by NNICE (y-axis) on the previously unseen 1000 simulated pseudo-bulk GEPs. Each panel shows results from (**A**) average across all quantiles; (**B**) each quantile; and (**C**) each cell type. Solid lines show simple linear regression lines for each quantile or cell type.

First, the performance of NNICE after training on 10,000 pseudo-bulk GEPs was evaluated. Convergence was reached for simulated pseudo-bulk within around 10 epochs (Fig. 6). For most cell types except CD4^+^ T cell, NNICE was able to provide estimates with well over 90% correlation on truth values (Table 3; Fig. 7). Estimates for CD4^+^ T cell was the lowest at *R* = 0.776 for training set and *R* = 0.797 for validation set. Performance metrics were similar on training and validation data, which indicated that the model showed no performance deficits due to overfitting on the training set, at least on the simulated pseudo-bulk dataset. Figure 7 shows performance on previously unseen simulated pseudo-bulk data (*n* = 1000) by quantiles, by cell types, and after averaging estimates across the five quantiles.

**Table 3:** Performance of NNICE on validation pseudo-bulk GEPs.

Cell type	Loss^a^	*R*	RMSE
B cell	0.0496	0.9104	0.1819
CD4^+^ T cell	0.1005	0.7973	0.1809
CD8^+^ T cell	0.0347	0.9543	0.2787
NK cell	0.0204	0.9833	0.2533
Myeloid	0.0139	0.9939	0.2180
Other	0.0140	0.9897	0.1842

^a^Loss is calculated by weighted sum of *R* and RMSE.

**Figure 8:**
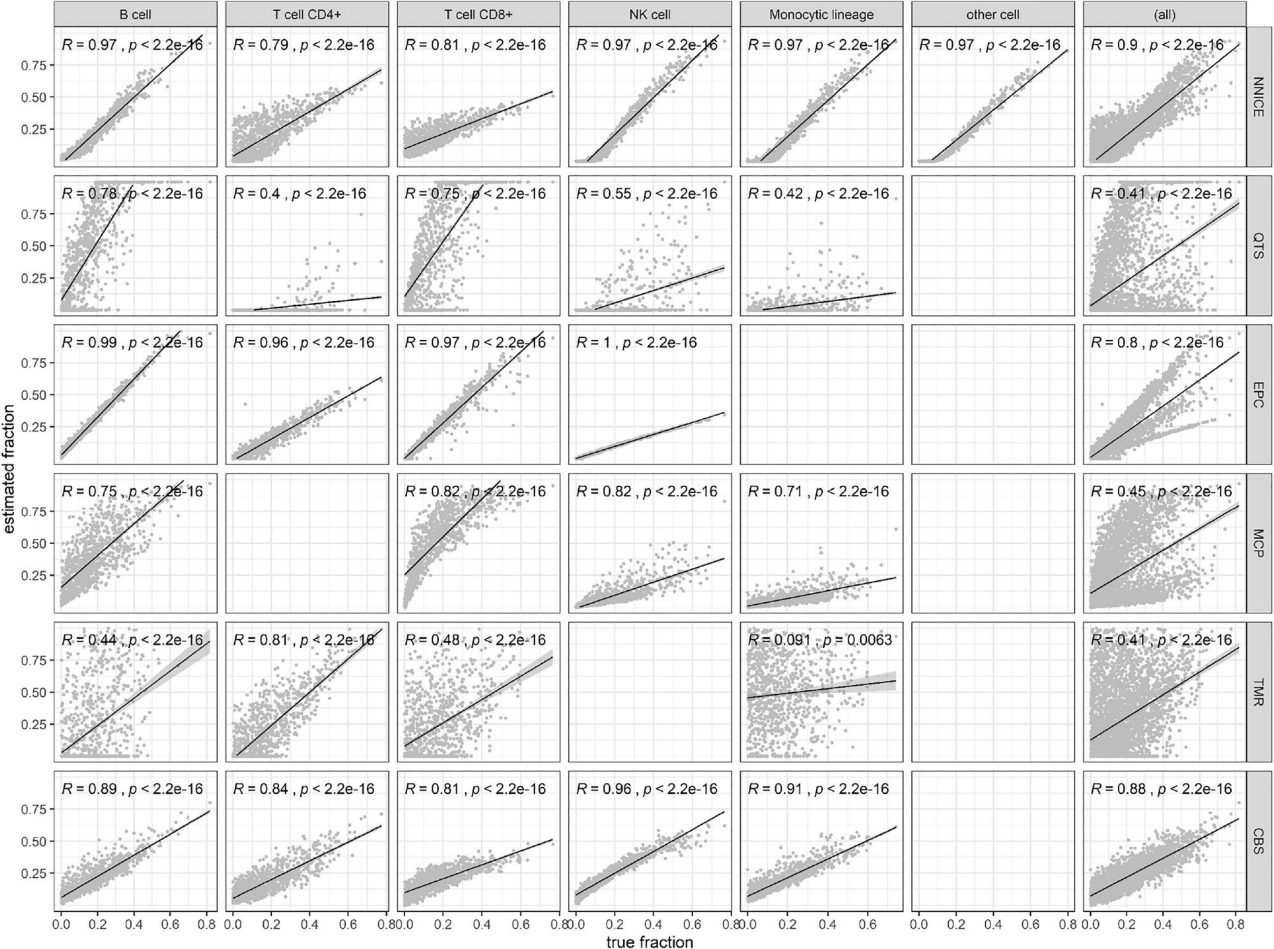
Comparison of prediction performance of NNICE and existing expression deconvolution methods on previously unseen 1000 pseudo-bulk GEPs. Each row shows results from a single method from the following: NNICE (trained on simulated data); QTS (quanTIseq); EPC (EPIC); MCP (MCP-counter); TMR (TIMER); and CBS (CIBERSORT). Each column represents results for a single cell type from the following list: B cell, CD4^+^ T cell, CD8^+^ T cell, NK cell, cells of monocytic lineage, other cells, and all cell types combined. For each scatter plot, x-axis is the true fraction and the y-axis is the estimated fraction by the methods. Blank plots indicate that the specific method provides no estimates for the particular cell type. Solid black line shows the computed Pearson correlation (R) between the true and estimated fractions between − 1 and 1 with p-value showing probability that the correlation in data is due to chance.

The performance of NNICE was compared against existing methods on previously unseen 1000 pseudo-bulk GEPs; refer to Fig. 8. NNICE trained on 10,000 pseudo-bulk GEPs showed the best performance among all existing methods at overall Pearson correlation *R* = 0.9. The second-best performing tool on pseudo-bulk GEP was CIBSERSORT at *R* = 0.88.

### Evaluation of model trained on real bulk tissue GEPs with FACS-quantified cell type proportions

**Figure 9:**
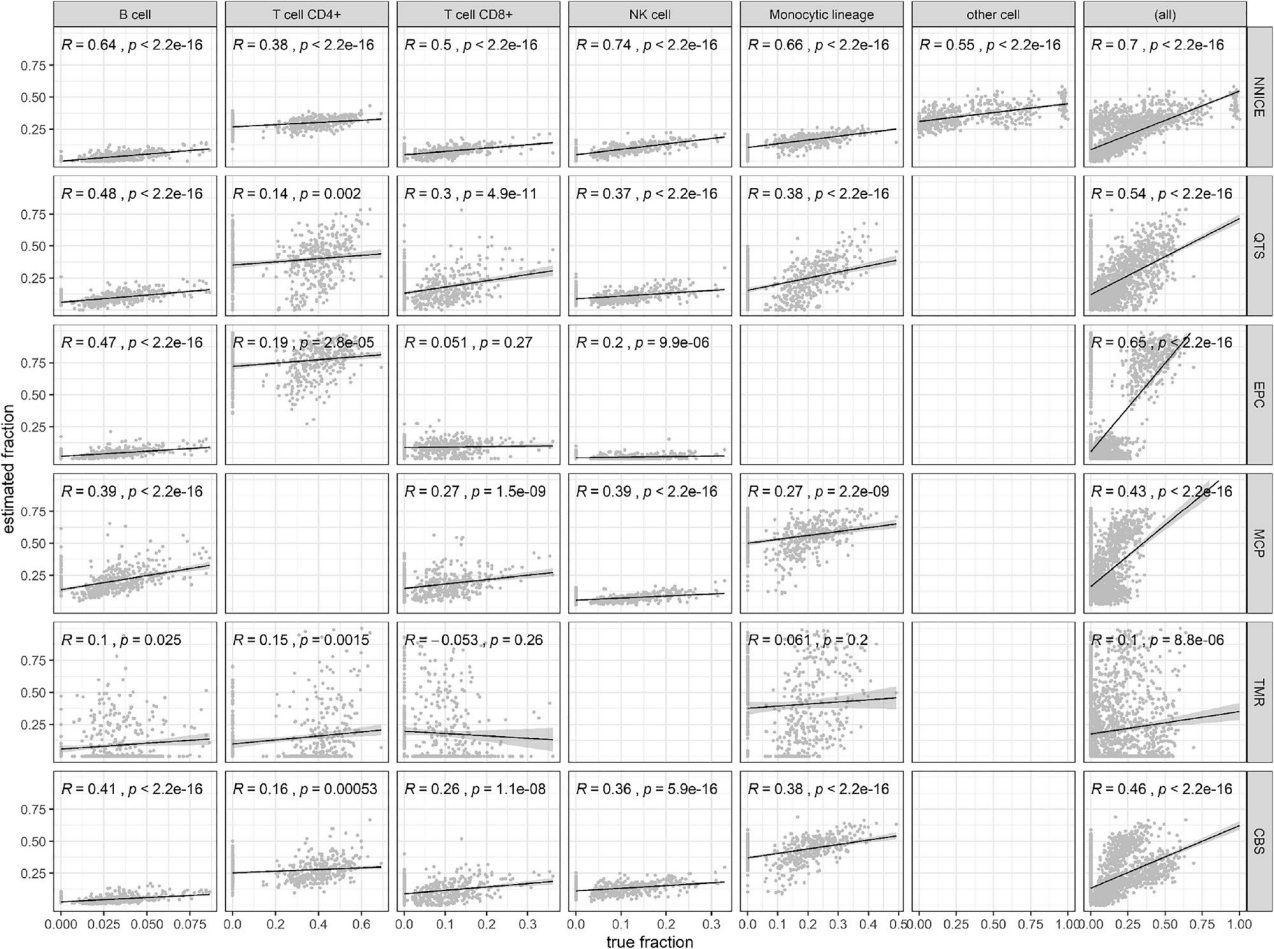
Comparison of prediction performance of NNICE and existing expression deconvolution methods on previously unseen 468 GEPs from SDY67 dataset. Each row shows results from a single method from the following: NNICE (trained on real data); QTS (quanTIseq); EPC (EPIC); MCP (MCP-counter); TMR (TIMER); and CBS (CIBERSORT). Each column represents results for a single cell type from the following list: B cell, CD4^+^ T cell, CD8^+^ T cell, NK cell, cells of monocytic lineage, other cells, and all cell types combined. For each scatter plot, x-axis is the true fraction and the y-axis is the estimated fraction by the methods. Blank plots indicate that the specific method provides no estimates for the particular cell type. Solid black line shows the computed Pearson correlation (R) between the true and estimated fractions between − 1 and 1 with p-value showing probability that the correlation in data is due to chance.

To evaluate the performance of NNICE trained with real data, tenfold cross-validation approach was used to train on the available 468 real bulk tissue GEP from the SDY67 dataset to evaluate its performance. Figure 9 shows prediction results from tenfold cross-validation of NNICE on SDY67 dataset. Correlation between the predicted and true fractions was similar across the 5 quantiles but the model did not perform consistently across all six cell types. Estimates from NNICE on SDY67 dataset was most accurate on the NK cells (*R* = 0.745) and least on the CD4^+^ T cells (*R* = 0.3833). Across all cell types, however, predictions from the NNICE model showed greater correlation with truth (*R* = 0.7) when compared with existing methods, of which predictions from EPIC had the highest correlation (*R* = 0.65); refer to Fig. 9. Table 4 shows a summary of overall performance of all expression deconvolution tools compared in this study on the two datasets.

**Table 4:** Benchmark results of the cascade oscillators model.

Tool	Simulated (*n* = 1000)	SDY67 (*n* = 468)
NNICE	**0.9**	**0.7**
quanTIseq	0.41	0.54
EPIC	0.8	0.65
MCP-counter	0.45	0.43
TIMER	0.41	0.1
CIBERSORT	0.88	0.46

Best performances are highlighted in bold.

## Discussion

Enormous success of deep learning in the fields of computer vision and natural language processing has prompted their application to various problems in computational biology over the recent decade. Because ANN models have shown to outperform many of the existing algorithms, it was reasonable to expect potential improvements over existing methods of expression deconvolution by applying ANN models. This research endeavor, however, was met with numerous challenges.

Firstly, ANNs usually require massive amounts of data to train, in orders of thousands if not millions, depending on the type of problem. It is not impossible to train on a smaller amount of data; however, ANNs often fail to achieve convergence with insufficient training data during training, leading to unstable predictions. In order to overcome this limitation, it was proposed that models would be trained on artificial gene expression data simulated from scRNA-seq datasets. This way, an arbitrarily large amount of pseudo-bulk GEPs could be generated to optimize performance of ANN models. Using simulated GEPs to benchmark and validate tools for expression deconvolution is not a novel idea—several groups have previously simulated pseudo-bulk GEP data from both RNA-seq^13,15^ and scRNA-seq^9,10^. Pseudo-bulk simulation involves the following steps: (1) simulation of cell type fractions within each pseudo-bulk mixture; (2) subsampling of single cell GEPs or reads from bulk GEPs in proportions simulated in the first step; and (3) summation of reads across all features up to a maximum cell or read count. Although this simulation approach is often used, previous studies have focused on validating performance of expression deconvolution tools on these datasets; therefore, validation of the simulation approach itself was largely neglected. Artificial datasets simulated in this way may be poor representations of real data because bulk-RNA-seq profiles are not simple summations of scRNA-seq profiles that have been subject to many technical biases and errors, for example, drop-outs. This could be one of the reasons underlying poor performance of NNICE on unseen real or simulated data. There is a need for existing pseudo-bulk GEP simulation strategies to undergo rigorous evaluations to assess to what extent simulated datasets resemble real datasets. This way, new algorithms should be developed to generate simulated datasets that are increasingly similar to real datasets. Computational approach to generate new data is resource-efficient; however, large-scale studies that involves collection of new gene expression and cell fractions data would also help overcome the current shortage of training data with known cell type proportions data.

Second, underfitting and overfitting are two common problems in machine learning. In particular, preventing overfitting was a major challenge for this project because in the end, none of the neural network models were able to provide stable and robust estimates for tissue immune contexture across the three datasets considered (simulated, and SDY67). Models trained only on simulated pseudo-bulk GEP data failed to show robust performances when applied to GEP from real bulk tissues (SDY67). Since previous works that applied deep learning framework to expression deconvolution used simple densely connected layers, the model architectures tested in this project were mostly also variations of dense neural networks. Therefore, there it is necessary to test whether different model architectures and optimization strategies have a positive impact on the performance of expression deconvolution and generalizability of the model to unseen data.

Another observation from our experiment results is that the model outputs are quite different for different quantiles. A unified framework on how to extract useful information on this uncertainty need to be further explored. Developing such a framework would require additional strategies and evaluations that are beyond the scope of this study. In our study, we averaged the outputs from all quantiles to obtain a less biased estimate. This averaging method is a simple way to mitigate the variations observed across different quantiles and provides a more stable estimate. We understand that this might not fully address all concerns related to uncertainty. In future research, a more comprehensive approach could involve evaluating the performance of individual quantiles and determining specific criteria for selecting the most reliable quantiles based on the application context. Additionally, further investigations could explore advanced methods for combining quantile estimates in a way that more accurately reflects the underlying data distribution and uncertainty.

There is a critical concern regarding the evaluation metrics used in our study, specifically the reliance on Pearson correlation coefficients to assess model performance. While Pearson correlation coefficients capture the correlation of trends between predicted and true fractions, they can be misleading if the regression slope is significantly different from 1 or the intercept is not zero. This issue is evident in some cases in Fig. 8, where, despite high correlation coefficients (e.g., R = 1), the model consistently underestimates or overestimates the true fraction due to non-ideal slopes and intercepts. We acknowledge this limitation and agree that it is crucial to consider the slope and intercept coefficients for a more accurate evaluation of model performance. Although our NNICE model generally showed reasonable slopes and intercepts, particularly in the first row of Fig. 8, we observed that for CD4^+^ and CD8^+^ T cells, the TMR and EPC models had better slopes and intercepts. While we did not include a detailed analysis of these metrics in the current study, we recognize their importance and plan to incorporate such an analysis in future research. This will help provide a more comprehensive assessment of the model's accuracy and reliability in estimating cell type fractions. Another observation is that the model accuracy for CD4^+^ T cells is significantly worse than other cell types. Upon some literature review, we recognize that CD4^+^ T cells exhibit a high degree of functional plasticity and heterogeneity^25^. This variability can lead to inconsistencies in model performance. The current model architecture may not be complex enough to capture the nuanced behaviors of CD4^+^ T cells. Exploring more sophisticated models or algorithms could potentially enhance accuracy in the future.

With all things considered, it is difficult to conclude that estimates for immune cell type fractions by NNICE models trained on either simulated or real data are true and accurate. Although the estimates should not be interpreted as ground-truth cell type fractions within tissues, it is also important to recognize that these values have potential to serve as intermediate risk scores for prognostic use, while providing additional insight into the immune contexture within the bulk tissues.

The proposed NNICE model was able to successfully recover ground-truth cell type fraction values given unseen bulk mixture gene expression profiles from the same dataset it was trained on. NNICE was not sufficiently robust to provide consistent performance across different datasets; however, this could be resolved either with acquisition of additional good quality bulk gene expression datasets or improvements to simulation algorithms that generate pseudo-bulk profile data.

## Data Availability

NNICE is available at https://github.com/ywjin0707/NNICE. Bulk mRNA-seq (single-end Illumina HiSeq 2000) gene expression data from 468 whole blood samples obtained from healthy participants aged between 50 and 74 was downloaded from the ImmPort database (accession SDY67 https://www.immport.org/shared/study/SDY67). scRNA-seq data from immune cells isolated by fluorescence-activated cell sorting was downloaded from the Zheng et al.’s publication https://github.com/10XGenomics/single-cell-3prime-paper.
